# Resolving the evolution of the mammalian middle ear using Bayesian inference

**DOI:** 10.1186/s12983-016-0171-z

**Published:** 2016-08-24

**Authors:** Héctor E. Ramírez-Chaves, Vera Weisbecker, Stephen Wroe, Matthew J. Phillips

**Affiliations:** 1 University of Queensland, School of Biological Sciences, Goddard Building 8 St. Lucia, Brisbane, QLD 4072 Australia; 2 Division of Zoology, School of Environmental and Rural Sciences, University of New England, Armidale, NSW 2351 Australia; 3 School of Earth, Environmental and Biological Sciences, Queensland University of Technology, Brisbane, QLD 4000 Australia

**Keywords:** Australosphenida, Middle ear detachment, Meckel’s groove, Postdentary trough, Theria

## Abstract

**Background:**

The minute, finely-tuned ear ossicles of mammals arose through a spectacular evolutionary transformation from their origins as a load-bearing jaw joint. This involved detachment from the postdentary trough of the mandible, and final separation from the dentary through resorption of Meckel’s cartilage. Recent parsimony analyses of modern and fossil mammals imply up to seven independent postdentary trough losses or even reversals, which is unexpected given the complexity of these transformations. Here we employ the first model-based, probabilistic analysis of the evolution of the definitive mammalian middle ear, supported by virtual 3D erosion simulations to assess for potential fossil preservation artifacts.

**Results:**

Our results support a simple, biologically plausible scenario without reversals. The middle ear bones detach from the postdentary trough only twice among mammals, once each in the ancestors of therians and monotremes. Disappearance of Meckel’s cartilage occurred independently in numerous lineages from the Late Jurassic to the Late Cretaceous. This final separation is recapitulated during early development of extant mammals, while the earlier-occurring disappearance of a postdentary trough is not.

**Conclusions:**

Our results therefore suggest a developmentally congruent and directional two-step scenario, in which the parallel uncoupling of the auditory and feeding systems in northern and southern hemisphere mammals underpinned further specialization in both lineages. Until ~168 Ma, all known mammals retained attached middle ear bones, yet all groups that diversified from ~163 Ma onwards had lost the postdentary trough, emphasizing the adaptive significance of this transformation.

**Electronic supplementary material:**

The online version of this article (doi:10.1186/s12983-016-0171-z) contains supplementary material, which is available to authorized users.

## Background

**Fig. 1:**
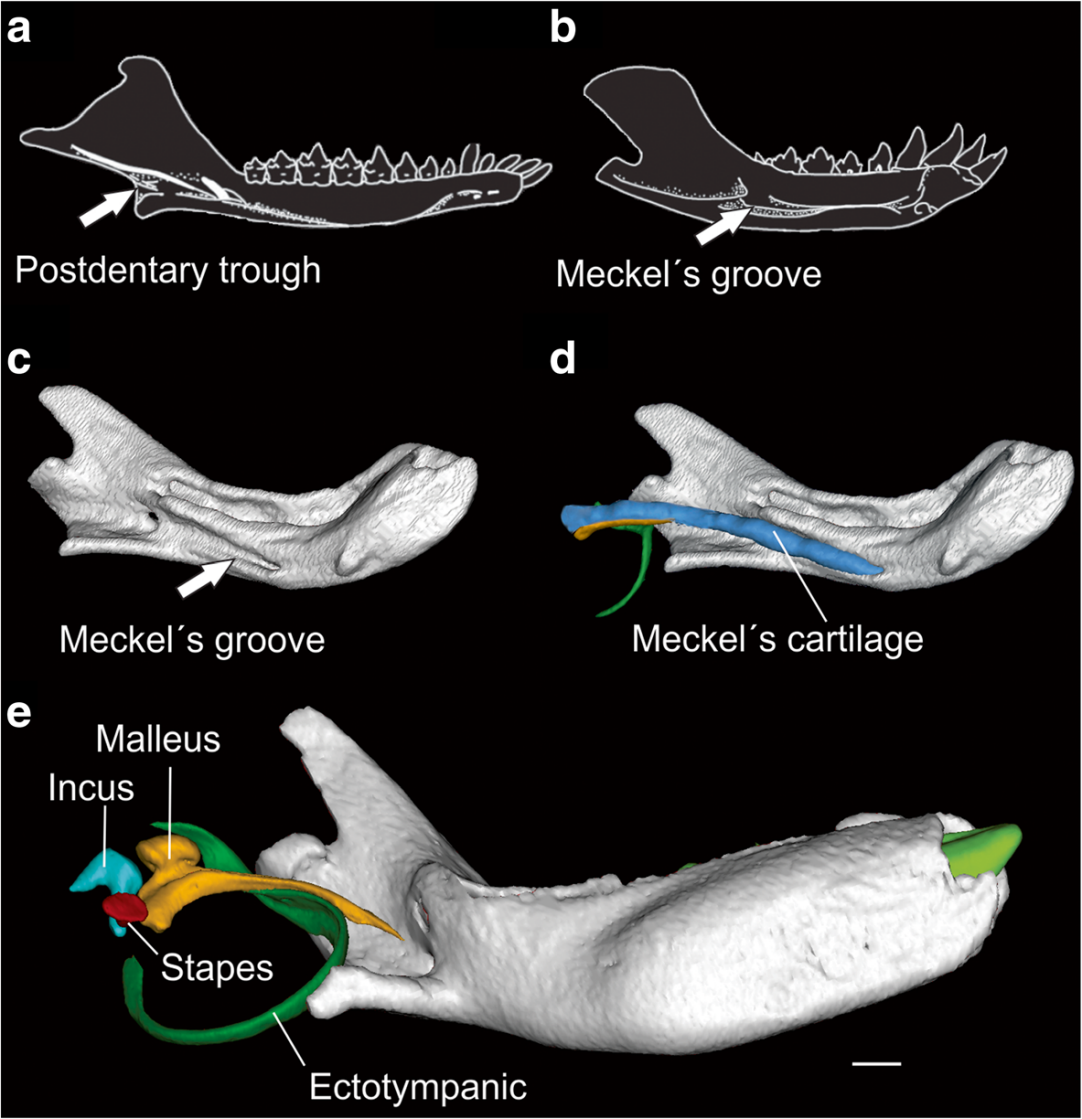
Dentary of **a**) *Morganucodon* with a mandibular middle ear based on the presence of a postdentary trough (redrawn from [48]); **b** Dentary of *Liaoconodon* with a partial mammalian middle ear based on the presence of Meckel’s groove (redrawn from [9]; **c**–**e** Dentary of the woylie, *Bettongia penicillata* at different developmental stages. c) presence of Meckel’s groove; **d** Meckel’s groove filled with the Meckel’s cartilage connecting the malleus to the dentary – this stage recapitulates or is similar to the partial mammalian middle ear found in some Cenozoic mammals; **e** individual showing a closed Meckel’s groove and absence of the connection, and hence, representing the definitive mammalian middle ear. The postdentary trough is not recapitulated during development in extant mammals. Orange, malleus; green, ectotympanic; blue, Meckel’s cartilage; light blue, incus; red, stapes; light green, incisor. Scale bar: 1 mm

One of the most famous and complicated transformations in vertebrate evolution is the origin of the mammalian middle ear bones (ectotympanic, incus and malleus) from load-bearing post-dentary elements (angular, articular and quadrate) during the evolution of synapsids [1]. This is also the oldest and best-documented example of developmental recapitulation of an evolutionary transformation [2–5], as the final separation of the middle ear from the dentary through disappearance of Meckel’s cartilage occurs in mammalian development as in evolution [6, 7] (Fig. 1).

The freely suspended middle ear of extant adult mammals is considered a derived pattern termed the definitive mammalian middle ear (DMME) [8]. A DMME has also been reported in other extinct mammal lineages, including multituberculates and several cladotheres (close therian relatives) [9]. Although a detached middle ear has been considered a defining feature of living mammals [10, 11], there is mounting evidence that the DMME was in fact acquired independently in monotremes and therians [7, 10, 12–15], which suggests strong selection for a sensitive auditory system adapted to high-frequency sounds [16, 17]. However, current hypotheses on the convergent evolution of middle ear bones are complex and controversial, partly because of a lack of phylogenetic resolution and partly because the interpretation of the fossil evidence is difficult [7, 18].

Some clues as to the sequence in which the mammalian middle ear evolved come from two additional patterns of the mammalian middle ear in the fossil record [7]. Both of these include a permanent connection of the middle ear bones to the dentary. The most plesiomorphic is the mandibular middle ear of cynodonts (MMEC; Fig. 1a), in which the middle ear bones are fully attached to the posterior part of the dentary, and are housed in a postdentary trough and angular fossa. In contrast, the “partial mammalian middle ear” (PMME; Fig. 1b), also termed “transitional mammalian middle ear” [9], has the middle ear bones connected to the dentary by an ossified or possibly cartilaginous Meckel’s cartilage [6, 7, 9, 19]. In the PMME, the ectotympanic ring and the malleus have no direct contact with the mandible, which therefore lacks a postdentary trough and angular fossa [20]. The PMME has been observed in eutriconodont and spalacotheroid mammals. It is also expected more generally across fossil mammals that lack a postdentary trough, but retain a prominent Meckel’s groove [6, 19]. The PMME is found in early stages of middle ear development in extant mammals (Fig. 1c), thus reinforcing the impression that it represents an intermediate evolutionary condition [6].

Hypotheses of independent acquisitions of the DMME are based on (1) differences between monotremes and therians regarding the origins of the jaw-opening muscles [13]; (2) hypotheses of a retention of the angular, articular and prearticular bones directly attached to the lower jaw in Cretaceous monotremes (particularly *Teinolophos trusleri*), inferred from the suspected presence of a postdentary trough to accommodate these bones, and (3) the polyphyletic distribution of the DMME across Mesozoic mammals, as inferred from the absence of a postdentary trough and Meckel’s groove.

**Fig. 2:**
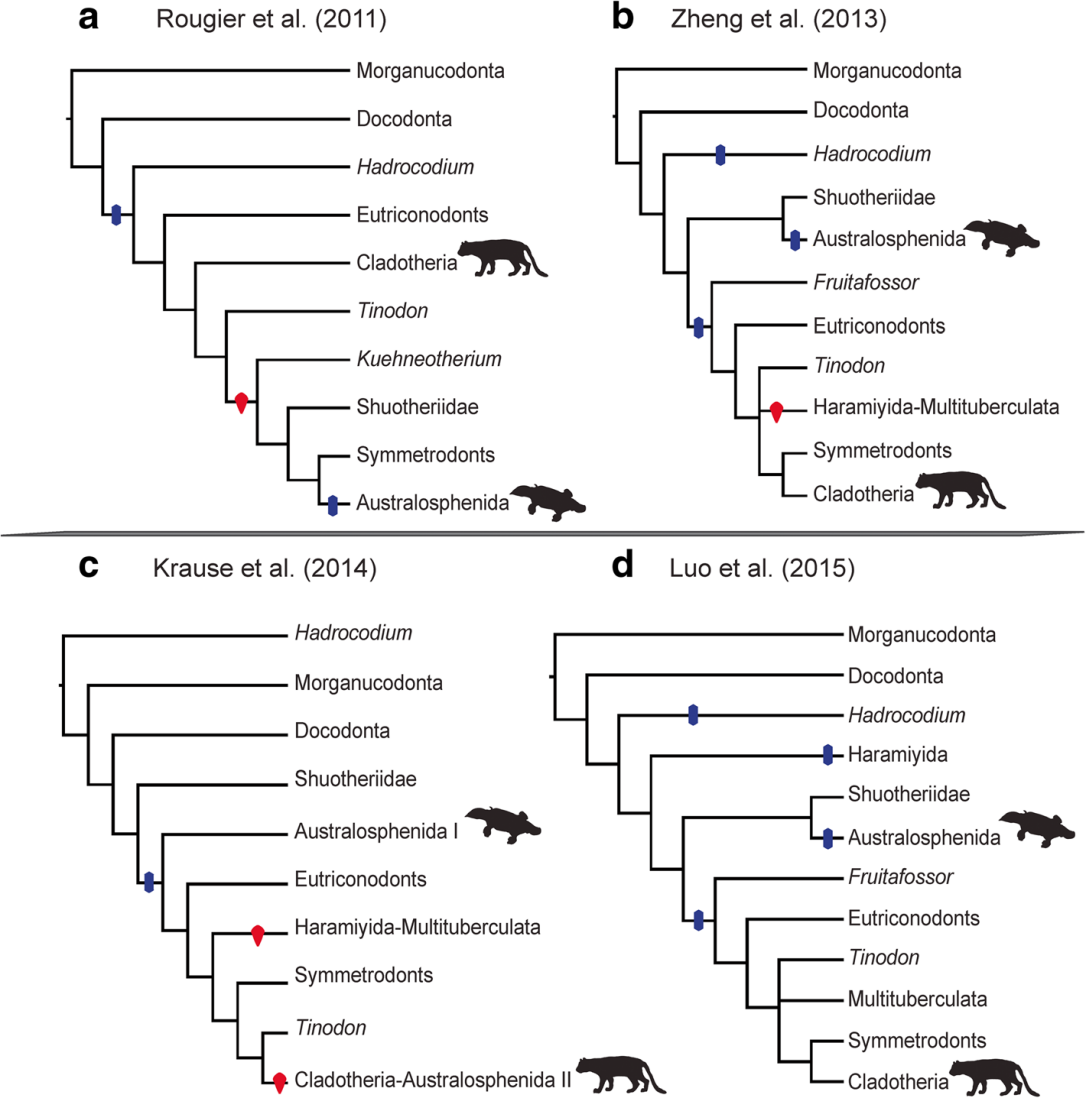
A summary of parsimony inference for postdentary trough transformation based on four recent phylogenies [21–24] with varying character coding. The reconstructions minimize the number of postdentary trough losses (blue) and regains (red). Note that taxon sampling differs between the studies (**a**-**d**). Placements of monotreme and therian mammals are represented by the platypus and ocelot, respectively. In Krause et al. [23] the symbols I and II indicate independent origins for australosphenidian taxa

Unfortunately, the historical emphasis on whether middle ear bone migration occurred independently in therian and monotreme ancestors has often overshadowed a broader taxonomic perspective on this evolutionary process. Phylogenetic and coding variation among recent studies allow for up to seven such independent migrations, based on the loss of the postdentary trough, along lineages leading to monotremes, euharamiyidans, multutuberculates, *Hadrocodium*, *Fruitafossor*, eutriconodonts, and trechnotheres (derived symmetrodonts and cladotheres). In Fig. 2 we provide a summary of parsimony inference for postdentary trough transformations based on four recent phylogenies [21–24] with varying taxonomic composition and character coding. Most remarkably, in three of these analyses the parsimony solution requires one or two reversals back to postdentary attached middle ear bones. Only the phylogeny of the most recent dataset [22] unambiguously supports losses of the postdentary trough (four in total), although that study did not include the basal symmetrodont *Kuehneotherium* which could add an extra loss of the postdentary trough connection, depending to its phylogenetic position.

Each of the proposed independent origins of the DMME is to some extent controversial or ambiguous. In some cases the phylogeny is controversial, such as for the relationships of haramiyidans (e.g. [22, 25]). Character coding is also contentious. Notably, there has been increasing debate about the apparent absence of a postdentary trough in the early mammaliaform *Hadrocodium*, which now appears to be a juvenile specimen with limited preservation of poorly ossified areas having erased evidence of a postdentary trough [20, 26–28]. The controversies surrounding character coding and preservation artifacts also affect the interpretation of key Cretaceous fossil monotremes. There has been a long debate regarding the presence of a postdentary trough and Meckel’s groove in *Teinolophos* and *Steropodon* [14, 18, 29, 30]. Comprehensive reviews now tend to support the absence of a postdentary trough from both species [18, 29, 30]. The suggested Meckel’s groove in *Steropodon* has been alternatively explained as “pre-depositional breakage” of the only known specimen [31].

Historically, two main alternative scenarios [19] have been described for the apparent multiple origins of the DMME during evolution: (1) the DMME was present in the common ancestor of Mammalia, but some clades (eutriconodonts and spalacotheroids) re-evolved a Meckel’s cartilage middle ear attachment to the mandible through paedomorphosis, and hence a PMME; or (2) the DMME was absent in the mammalian common ancestor and evolved once in extant monotremes, for a second time in multituberculates, and again in the ancestors of both marsupials and placentals [7, 19]. The latter scenario does not clearly identify whether the common ancestor of modern mammals had a PMME or a MMEC, and resolution of these paths towards the DMME depends on phylogenetic relationships and the status of characters in several fossil taxa [7].

As noted, both of the above scenarios are partially based on controversial evidence and have also exclusively relied on parsimony analyses that minimize rather than estimate the number of transformations. Here, we infer the phylogenetic history of middle ear migration in mammals, and test which scenario better explains the evolution of the DMME, using Bayesian methods to reconstruct the ancestral states of the two key characters associated with middle ear detachment. We also revisit the possibility of preservational artifacts influencing the scoring of Meckel’s groove and the postdentary trough in morphological matrices, using simulations of erosions in virtual 3D models of monotreme lower jaws.

## Results

### Virtual reconstruction of possible diagenetic artifacts and character re-scoring for the postdentary trough and Meckel’s groove

**Fig. 3:**
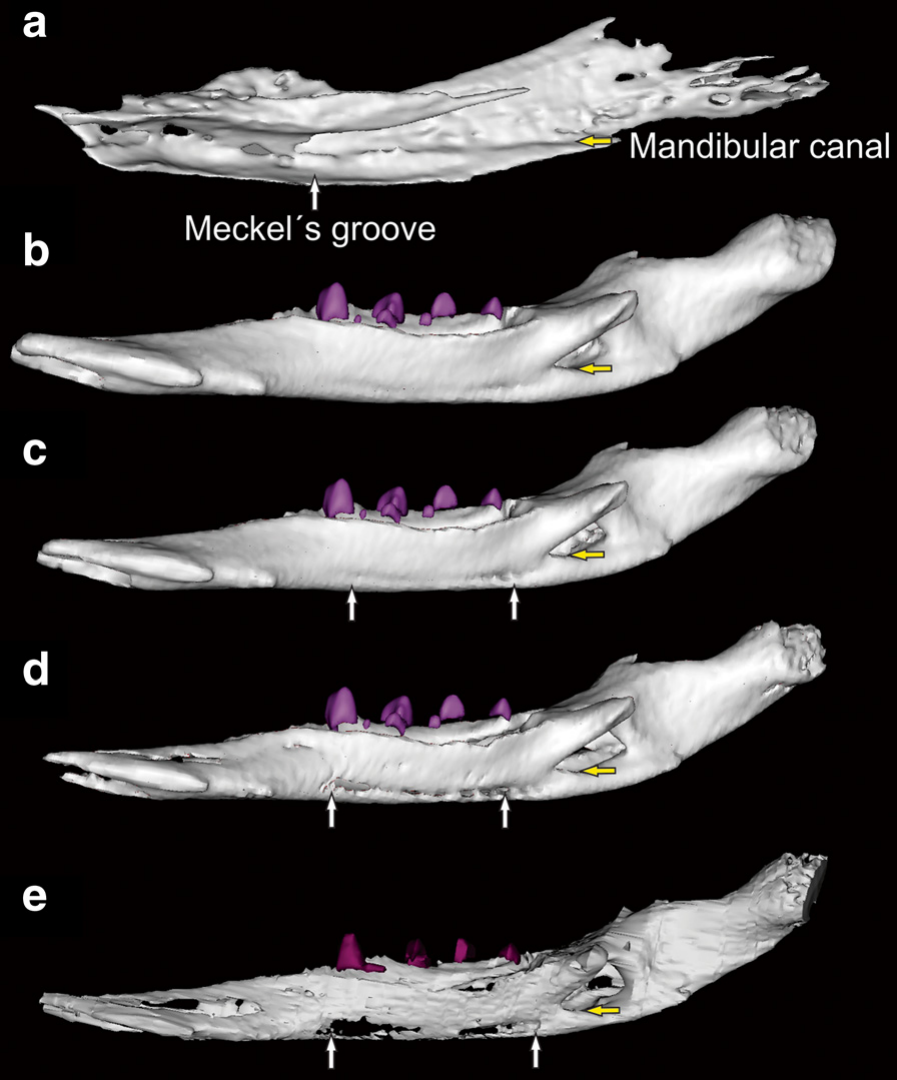
Lingual view of the developing and artificially eroded platypus dentary. In early development of the platypus (**a**) Meckel’s cartilage lies in a deep Meckel’s groove. In a juvenile specimen with Meckel’s groove closed (**b**), a faint seam on the ventral border of the mandibular canal is present along the length of the dentary. Through relatively minor virtual erosion of the specimen (**c**–**e**), this trace is exaggerated into a structure resembling a Meckel’s groove while the detailed topology of the dentary is otherwise retained. Coding Meckel’s groove as present only where it is unambiguously well-developed (see Methods) therefore appears to be the best strategy

We found that the presence of Meckel’s groove and its disappearance can be traced in development for the platypus and echidna (see also [6]). However, we could not identify any developmental evidence for the presence of a postdentary trough at any stage. Both our virtual erosion and thresholding of 3D reconstructions of a juvenile platypus dentary with recently detached middle ears showed that an anatomical feature resembling a substantial Meckel’s groove could be easily produced from a slight lingual indentation (Fig. 3d and e). This indentation could be a filled-in trace of Meckel’s groove, or a ventral “lip” below the developing mandibular canal (Fig. 3a). Notably, except for our most extreme erosion scenario (Fig. 3e), this artifact could be produced without compromising the anatomical detail in other parts of the dentary, suggesting that erosion of a dentary in the fossil record might not be noticed. This suggests that the coding for the degree of development of Meckel’s groove (0: Well developed; 1: Weakly developed; 2: Vestigial or absent) that has been used as a character in phylogenetic analyses may in practice be difficult to distinguish based on sub-optimally preserved, eroded fossils, especially for the state ‘weakly developed’ and ‘vestigial or absent’. Using this observation we re-scored Meckel’s groove in both datasets as: (0) Meckel’s groove developed (including former character state 1: “weakly developed”), (1) Meckel’s groove not developed or vestigial. A developed Meckel’s groove, in this case, is taken as evidence of a PMME. Using this observation and based on new fossil information [21, 29, 30], we re-coded the presence/absence of the postdentary trough in *Hadrocodium* and several australosphenidans (*Ausktribosphenos*, *Bishops*, *Steropodon*, and *Teinolophos*). These recoded datasets were then used for the phylogenetic analyses to trace the origins of the DMME.

In the first dataset [22], all euharamiyidans, multitubeculates and cimolodontans were re-scored as Meckel’s groove “not developed or vestigial”. For eutriconodonts, spalacotheroids, and cladotheres that were coded as Meckel’s groove “Weakly developed” (see [22]), this character was re-coded as “Meckel’s groove developed” in our analyses. Similarly, Meckel’s groove was re-coded for eutriconodonts and cladotheres in the second dataset [21] to match our re-coding for the first dataset [22].

For *Hadrocodium*, the postdentary trough has been scored as “absent”, and Meckel’s groove as “weakly developed” [21] or “vestigial or absent” [22] respectively. These characters were coded as “present” and “developed” respectively in our analyses based on new evidence that suggests *Hadrocodium* is a juvenile specimen with limited preservation of poorly ossified areas largely erasing evidence of its postdentary trough [20, 26–28], and Meckel’s groove.

For *Asfaltomylos* we scored the postdentary trough as “present”, and Meckel’s groove as “uncertain”. Although Meckel’s groove has been coded as “vestigial or absent” [22], and as “well developed” [21], this character cannot be observed in the only known specimen in which this region of the dentary is damaged [32]. This new interpretation also changes the codification of the curvature of Meckel’s sulcus as “uncertain”. Postdentary trough was coded as “present” in our analyses.

For *Ausktribosphenos*, the postdentary trough has been considered as “present” [33], or “absent” [21], and Meckel’s groove as “well developed”. The dentary of *Ausktribosphenos* has been illustrated including a remnant of Meckel’s groove that extends until the m3 [34]. However, similar remnants were observed when thresholding was applied to virtually reconstructed models of juvenile platypus (*O. anatinus*) to simulate removal of layers of bone, to mimic diagenetic bone attrition (Fig. 3). The postdentary trough seems to be absent based on one illustration (see [34]). We concurred with the interpretation of the postdentary trough as “absent” [21], but recoded Meckel’s groove as “developed”, based on our virtual reconstructions and thresholding comparison of a juvenile platypus that lost Meckel’s groove during development. This shows the possibility of the character in *Aukstribosphenos* being an artefact. In an alternative analysis the postdentary trough was coded as “uncertain”, but the topologies of the trees and posterior probabilities were not affected (Additional file 1: Table S1).

For *Bishops*, we concurred with the interpretation for scoring the postdentary trough as “absent” [21], but we considered Meckel’s groove as “developed”. Although the postdentary trough has been coded as present based on previous datasets [22], the description of the illustrations of the taxon [35] show no trace of the presence of postdentary trough.

In *Steropodon*, the postdentary trough might be stated as “absent” and Meckel’s groove as “weakly developed” [21], contrary to recent datasets (see [22, 33]) in which these characters were coded as “present” and “well developed”, respectively. The illustration of the holotype of this taxon [31] shows no clear presence of a postdentary trough. Although the authors mentioned that a depression in the lingual side of the dentary extends until m3, they are unsure whether this represents a Meckel’s groove or pre-depositional breakage. We expect this feature results from “pre-depositional breakage”, but coded the character as “uncertain”. We scored the postdentary trough alternatively, as either “absent” or “uncertain” in our analyses.

For *Teinolophos* the postdentary trough has been considered “present” [14], or “absent” [21, 33]; and Meckel’s groove as “well developed” [22], or “weakly developed” [21]. We consider the postdentary trough to be “absent” and Meckel’s groove “developed” (following [29]). Based on our virtual reconstructions and because the postdentary trough is clearly identifiable in taxa for which this structure is present, we concur with *Teinolophos* lacking a postdentary trough.

### Phylogenetic ancestral state reconstructions

**Fig. 4:**
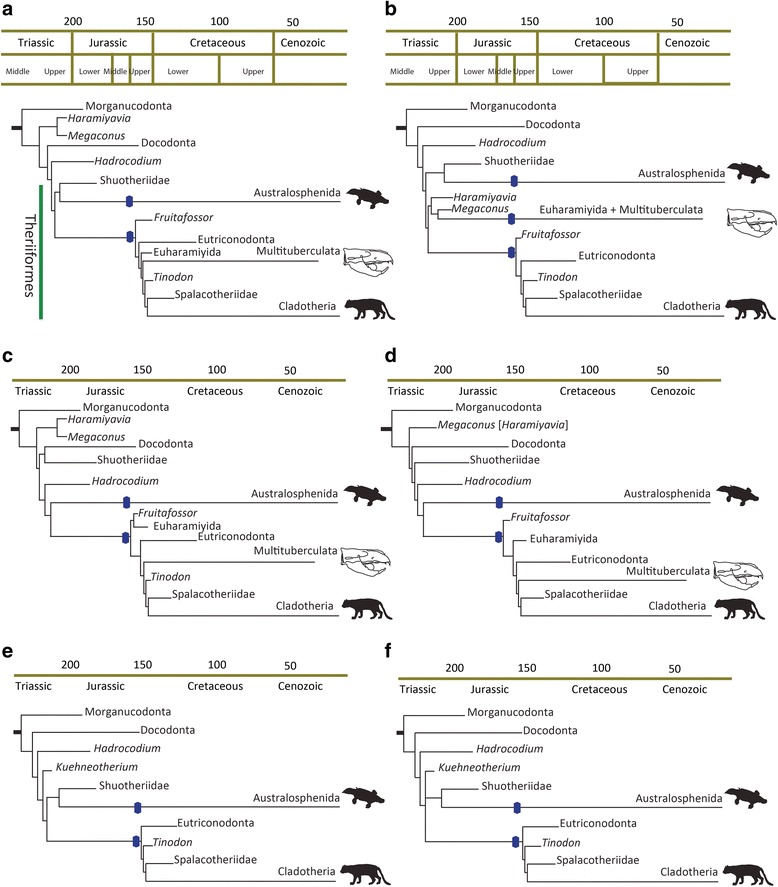
Bayesian inference of the loss of the postdentary trough among mammals (blue), based on the re-coded datasets (**a**–**d** [22]; **e**–**f** [21]). Each tree summarizes the following analyses: **a**, **b** Shuotheriidae unconstrained (allowed to group with australosphenidans); **b** constraining monophyly of the multicuspate haramiyidans and multituberculates; **c** constraining Shuotheriidae to fall outside Mammalia; **d** excluding cheek teeth characters; **e** forcing *Kuehneotherium* outside Mammalia; **f** allowing *Kuehneotherium* to fall freely in the phylogeny. Postdentary trough is lost twice in all the analyses except in (**b**) when the earliest haramiyidans (e.g *Haramiyavia*) are forced together with Euharamiyida and multituberculates. The analyses excluding cheek teeth characters (**d**) place primitive haramiyidans outside Mammalia, with euharamiyidans and multituberculates both falling on the therian stem lineage–negating any requirement for a third loss of the postdentary trough in mammals. In (**d**) *Haramiyavia* is not included but likely forms a clade with *Megaconus* as observed in several of these trees (not b)

Our Bayesian ancestral state reconstruction based on the first dataset [22] suggests that the postdentary trough was lost independently, once in australosphenidans, and again in the common ancestor of *Fruitafossor* and Theriiformes (eutriconodonts, euharamiyidans, multitubeculates, spalacotheriids, and cladotheres), regardless of the placement of the pseudotribosphenic shuotheriids (Fig. 4a and c). Only when all haramiyidans and multituberculates were forced together was the postdentary trough lost a third time (Fig. 4b), in the common ancestor of multituberculates and advanced haramyidans (euharamiyidans). The ancestral condition of the postdentary trough does not re-evolve on the tree after the postdentary bones are detached from the dentary and the postdentary trough is lost (Fig. 4). The Bayesian posterior probability (BPP) for the ancestor of australosphenidans retaining a postdentary trough (Additional file 1: Table S1) is high (BPP > 0.92), except when Shuotheriidae was forced outside Mammalia (~0.63), while being very low for the ancestor of stem and crown monotremes (BPP < 0.01). Similarly, the posterior probability of the ancestor of Mammalia possessing a postdentary trough was high (BPP > 0.85), except when Shuotheriidae was forced outside Mammalia (~0.59).

The analyses of the second dataset [21], which does not include multituberculates or haramiyidans, also favours the postdentary trough being lost independently only twice, once in monotremes and once in the common ancestor of Theriiformes, regardless of the position of *Kuehneotherium* (Fig. 4e and f). All analyses (Additional file 1: Table S1) support the ancestor of australosphenidans retaining a postdentary trough (BPP > 0.92), and the ancestor of monotremes lacking a postdentary trough (BPP = 0.99). The presence of a postdentary trough at the origin of crown mammals in this dataset was also high (BPP > 0.94) in all the analyses.

Meckel’s groove is more variable in analyses of both datasets, with a DMME evolving from a PMME several times since the earlier loss of the postdentary trough. Meckel’s groove is well developed in adults at the crown ancestor of mammals (BPP > 0.95), then is independently lost in adults of recent australosphenidans (crown monotremes), multituberculates, and in several cladotheres, including along the stem lineages of both marsupials and placentals. In all analyses the ancestor of australosphenidans (and the ancestor of stem monotremes) was found to have a developed Meckel’s groove during adulthood (BPP > 0.84) (Additional file 1: Table S1).

## Discussion

Our Bayesian analysis supports a simple, directional two-step scenario of mammalian middle ear evolution. The first step includes the departure of postdentary bones from the dentary to form a partial mammalian middle ear (PMME); this occurred convergently in the northern hemisphere ancestors of therians and the southern hemisphere ancestors of monotremes (Fig. 4). Second, the transition from a PMME to a definite mammalian middle ear (DMME) ocurred multiple times, including at least three cases of independent evolution within extant mammals (in monotremes, metatherians and eutherians), between the late Jurassic (~163 Ma) and the Late Cretaceous (~80 Ma). In addition, our results clarify the ancestral mammalian condition, suggesting that that the ancestor of all mammals possessed a plesiomorphic, fully attached mandibular middle ear of cynodonts (MMEC).

The parallel evolution of the MMEC to the transitional PMME in therians and monotremes appears to represent a continuation of a mammaliform trend towards reduced postdentary bones and dominance of the squamosal-dentary jaw articulation, thus uncoupling the auditory and feeding systems of the skull [36]. We show that this transformation only occurred twice, if basal haramiyidans and multituberculates are placed as members of the therian stem lineage as supported by our analyses excluding cheek teeth. This is a far simpler scenario than the up to seven losses and re-gains of the post-dentary trough in our parsimony-based reconstructions of recently published coding and phylogenies (Fig. 2). MMECs in taxa such as *Haramiyavia*, *Kuehneotherium* and *Shuotherium* are thus plesiomorphic retentions, and not reversals from the DMME or the PMME as suggested by previous studies [7, 9].

In addition, our ancestral state reconstruction substantially pushes back the inferred date of MMEC loss in the australosphenidans (monotreme ancestors). This was considered to have occurred relatively recently, in the common ancestor of living monotremes, while our analysis suggests that this transformation occurred at least 120 million years ago in the ancestors of the Australian australosphenids (monotremes and ausktribosphenids, and *Bishops*).

Intriguingly, our results suggest a fast transition from a MMEC to a PMME: all known mammals up until ~168 Ma retained fully attached middle ear bones, while all mammalian families that diversified from ~163 Ma onwards had lost the postdentary trough. The rapidity of the MMEC/PMME transition emphasizes the key adaptive significance that has been attributed to this transformation [37].

Our analyses unambiguously identify the PMME as a phylogenetic stage preceding the evolution of a DMME [9, 19], regardless of whether Meckel’s cartilage was cartilaginous or ossified (as observed as a possibly peramorphic trait [1, 6, 7, 19] in some adult eutriconodonts and spalacotheroids). This conclusion is surprisingly robust to the phylogenetic position and presence or absence of postdentary troughs in controversial taxa (e.g. *Hadrocodium* and *Teinolophos*), which have previously represented debated obstacles in the interpretation of mammalian middle ear evolution (see Introduction).

Our results demonstrate that Bayesian inference represents a powerful tool for inferring ancestral character states, especially in complicated cases such as the evolution of the mammalian middle ear. Bayesian ancestral state reconstruction is standard in analyses of extant taxa, but has rarely been used with the inclusion of fossils, which in turn offers the possibility to overcome extinction biases that affect inferences from extant taxa alone [38].

Aside from the more nuanced approach of modelling evolutionary processes compared to the binary decision-making of parsimony, Bayesian posterior probabilities can provide predictive power to inform character state assignments in debated fossils. This is perhaps best illustrated with the use of ancestral reconstruction to provide us with a prior probability for a postdentary trough in *Hadrocodium*, which until recently had been considered to have a DMME. Consistent with recent finds of a MMEC in this species, Bayesian inference analysis retrieved a high probability (BPP > 0.82) of MMEC presence.

The coding of presence or absence of Meckel’s cartilage has caused some controversy, particularly in *Hadrocodium* and australosphenidans such as *Teinolophos,* which appear to have a Meckel’s groove imprint (see [39]). Our virtual simulation of erosion showed that we could easily create artifacts that are near-identical to the suspected Meckel’s groove in *Steropodon*. Another issue is the possibility that the presence of Meckel’s groove in juvenile fossils might indicate a later time of detachment during development, rather than partial attachment of the middle ear to the dentary in adulthood. This suggests that the current coding of different states of Meckel’s groove (absent or vestigial/weakly developed/strongly developed) incurs the risk of overestimating the presence of Meckel’s groove and affecting the estimation of when the DMME evolved. Coding Meckel’s groove as present only where it is unambiguously well-developed (see Methods) therefore appears to be the best strategy.

## Conclusions

This study used Bayesian analysis to provide the first probabilistic evidence that the most recent common ancestor of mammals originally possessed a cynodont-like middle ear with a loadbearing function. Detachment from the postdentary trough and functional uncoupling of the auditory and feeding apparatus occurred independently in therian and monotreme ancestors. The final detachment of the middle ear through disappearance of Meckel’s cartilage was rapid in some lineages and long delayed in others, matching the overall pattern of rapid diversification of mammals during this time. Functional and ecological comparisons in lineages with different detachment timeframes might provide information as to whether detachment was driven by selective pressures [13, 40], or represents a side-effect of developmental change elsewhere [7].

## Methods

### Virtual reconstruction of possible diagenetic artifacts and character re-scoring

To test whether the apparent presence of a postdentary trough and a weakly developed or vestigial Meckel’s groove in stem monotremes (e. g. *Teinolophos*, *Steropodon*) might be artifacts of poor fossil preservation, we used μCT scans of the dentary and middle ear bones of a juvenile platypus with a detached middle ear, but faint scarring on the lingual side of the dentary (Fig. 3). We used the Materialise Mimics software digitally removed the outer bone layers of the 3D reconstructed models obtained from the μCT scans by increasing the thresholding values of the model mesh, and also using the “erosion” tool, which removes the external layer of “3D pixels” (voxels). This was to simulate a situation where diagenetic erosion of bone could produce an artifact that simulates the presence and degree of development of Meckel’s groove (Fig. 3) in fossils, as has been suggested for *Steropodon* (see Introduction). Based on the information from the virtual reconstructions and comparisons with new evidence available for stem fossil monotremes [29, 30] and *Hadrocodium* [25, 26, 28], we re-coded the states of the postdentary trough and Meckel’s groove for several taxa (see Results) in two morphological datasets [21, 22]. Each of these matrices has a different taxonomic focus, but together they cover all of the relevant, known lineages for evolution of the DMME. We also used these μCT scans (Fig. 3) to identify any developmental evidence for the presence of a postdentary trough in extant monotremes.

### Phylogenetic analyses

For both datasets [21, 22], we explored the ancestral condition of the two osteological characters that have mainly defined the debate on single *versus* multiple origins of the DMME: the postdentary trough and Meckel’s groove (see Introduction). We used Bayesian inference within MrBayes 3.2.4 [41, 42] to infer the phylogeny using each recoded dataset, and ancestral state reconstruction for the postdentary trough and Meckel’s groove characters. Two independent analyses were run on three Markov chains for 5,000,000 generations, with trees sampled every 5000 generations for the second dataset [21], and for 10,000,000 generations, with trees sampled every 10,000 generations for the first dataset [22]. These chain lengths ensured clade frequency convergence between runs and estimated sample sizes for substitution parameters were >100 (using Tracer v1.5 [43]).

Both matrices were analysed with five partitions: 1. mandibulodental, 2. postcranial, 3. cranial characters, 4. Meckel’s groove, and 5. the postdentary trough. The Mkv + gamma evolutionary model allows for different evolutionary rates among characters, and was employed for the multi-character partitions. The Mkv model was used for Meckel’s groove and the postdentary trough, given that rates across sites (e.g. gamma) models are redundant for single character partitions.

Although the first dataset [22] includes 114 taxa, we only included 84 of these in our analyses. We excluded highly incomplete taxa (<12.5% of the characters; e.g. *Albertatherium*, *Aegialodon*) because they potentially bias branch length estimation [44], and hence phylogeny and ancestral state reconstruction. It is important that rate models for middle ear and Meckel’s groove evolution reflect evolvability during the Mesozoic. So in addition, the 25 modern placentals and marsupials were also excluded, because canalisation in these groups during the Cenozoic has apparently limited the plasticity of these traits and their inclusion would artifactually deflate inferred evolutionary rates for tracing their history [45]. Many other taxa are also <50% complete, but are less concerning because they are largely complete for the anatomical region partitions in which they are included. Similarly, for the second matrix [21] we excluded 3 taxa, *Drescheratherium* (low completeness), the modern *Erinaceus*, and *Didelphis*.

Alternative ancestral state reconstructions for each of the matrices were performed to cover different phylogenetic possibilities that stem primarily from arguments over molar cusp pattern homologies (e.g. [24, 46]), which are correlated among many characters, and therefore, could strongly mislead phylogeny if they were incorrectly assigned. Of particular importance are controversial molar cusp homologies between australosphenidans, boreosphenidans and the pseudotribosphenic shuotheriids, and between multituberculates and haramiyidans. We note also that the evolutionary rate inferred for cheek tooth characters is 2.5-fold higher than among other anatomical partitions, and high evolutionary rates increase phylogenetic signal erosion, and hence, susceptibility to non-phylogenetic biases, such as functional correlations [47]. To check for phylogenetic anomalies we ran both datasets [21, 22] without cheek tooth characters (Fig. 4d; Additional file 1: Figure S1 and S2). For the first dataset. [22], the “pseudotribosphenic” shuotheriids fell outside crown mammals and placement of the enigmatic, fossorial *Fruitafossor* was clarified as grouping closer to therians than to monotremes (Fig. 4d; Additional file 1: Figure S2). The second dataset [21] did not include *Fruitafossor* or the controversial multituberculates and haramiyidans, but cheek tooth exclusion clarified the placement of the early “symmetrodont” *Kuehneotherium* outside the mammalian crown (Additional file 1: Figure S1).

In recognition of these findings and phylogenetic uncertainties, the ancestral state reconstructions were performed on the full set of characters with (1) Shuotheriidae allowed to fall freely within the phylogeny (e.g. with australosphenidans), (2) constraining Shuotheriidae to fall outside Mammalia, and (3) constraining monophyly of the multicuspate haramiyidans and multituberculates (e.g. [25]). For the second dataset [21], which does not include the multicuspate taxa, two analyses were performed: the first allows the unstable *Kuehneotherium* to fall freely in the phylogeny, while the second forces it outside Mammalia, in agreement with our analysis in which cheek teeth characters were excluded.

## Additional file

Additional file 1: Figure S1. Bayesian inference phylogeny using Rougier et al.’s [21] dataset when cheek tooth characters were excluded. This analysis clarified the placement of the early “symmetrodont” *Kuehneotherium* outside the mammalian crown. **Figure S2.** Bayesian inference phylogeny for Luo et al.’s [22] dataset, but with cheek tooth characters excluded from. The “pseudotribosphenic” shuotheriids (purple) fell outside crown mammals and the placement of the enigmatic, fossorial *Fruitafossor* (yellow) was clarified as grouping closer to therians than to monotremes (green). **Table S1.** Posterior probabilities. Description: A: Shuotheriidae allowed to group with australosphenidans. B: Constraining Shuotheriidae to fall outside Mammalia. C: Constraining monophyly of the multicuspate haramiyidans and multituberculates. D: Forces *Kuehneotherium* outside Mammalia. E: Allows the unstable *Kuehneotherium* to fall freely in the phylogeny. 1: Alternative analyses including *Aukstribosphenos* and *Steropodon* with postdentary trough coded as absent. (DOCX 231 kb)

## Abbreviations

BPP: Bayesian posterior probability
DMME: Definitive mammalian middle ear
MMEC: Mandibular middle ear of cynodonts
PMME: Partial mammalian middle ear

## References

[1] Maier W, Ruf I (2016). Evolution of the mammalian middle ear: a historical review. J Anat.

[2] Allin EF (1975). Evolution of the mammalian middle ear. J Morphol.

[3] Anthwal N, Joshi L, Tucker AS (2013). Evolution of the mammalian middle ear and jaw: adaptations and novel structures. J Anat.

[4] Takechi M, Kuratani S (2010). History of studies on mammalian middle ear evolution: A comparative morphological and developmental biology perspective. J Exp Zool Part B.

[5] Reichert C. Über die Visceralbogen der Wirbelthiere im Allgemeinen und deren Metamorphosen bei den Vögeln und Säugethieren. Archiv Anat Physiol Wiss Med. 1837:120–222.

[6] Ramírez-Chaves HE, Wroe SW, Selwood L, Hinds LA, Leigh C, Koyabu D, Kardjilov N, Weisbecker V (2016). Mammalian development does not recapitulate suspected key transformations in the evolutionary detachment of the mammalian middle ear. Proc R Soc B.

[7] Luo Z-X (2011). Developmental patterns in Mesozoic evolution of mammal ears. Annu Rev Ecol Evol S.

[8] Allin EF, Hopson JA, Webster DB, Fay RR, Popper AN (1992). Evolution of the auditory-system in Synapsida (mammal-like reptiles and primitive mammals) as seen in the fossil record. Evolutionary biology of hearing.

[9] Meng J, Wang YQ, Li CK (2011). Transitional mammalian middle ear from a new Cretaceous Jehol eutriconodont. Nature.

[10] Martin T, Luo ZX (2005). Homoplasy in the mammalian ear. Science.

[11] Meng J, Wyss AR (1995). Monotreme affinities and low-frequency hearing suggested by multituberculate ear. Nature.

[12] Fleischer G (1978). Evolutionary principles of the mammalian middle ear. Adv Anat Embry Cell Biol.

[13] Hopson JA (1966). The origin of the mammalian middle ear. Am Zool.

[14] Rich TH, Hopson JA, Musser AM, Flannery TF, Vickers-Rich P (2005). Independent origins of middle ear bones in monotremes and therians. Science.

[15] Tatarinov LP (2009). Probable diphyletic formation of the mammalian middle ear: Monotremata and Theria. Paleontol J.

[16] Rosowski JJ, Webster DB, Fay RR, Popper AN (1992). Hearing in transitional mammals: Predictions from the middle-ear. Anatomy and hearing capabilities of extant mammals. Evolutionary biology of hearing.

[17] Manley GA (1972). A review of some current concepts of the functional evolution of the ear in terrestrial vertebrates. Evolution.

[18] Rougier GW, Forasiepi AM, Martinelli AG (2005). Comment on “Independent origins of middle ear bones in monotremes and therians” (II). Science.

[19] Ji Q, Luo Z-X, Zhang XL, Yuan CX, Xu L (2009). Evolutionary development of the middle ear in Mesozoic therian mammals. Science.

[20] Wang YQ, Hu YM, Meng J, Li C (2001). An ossified Meckel’s cartilage in two cretaceous mammals and origin of the mammalian middle ear. Science.

[21] Rougier GW, Apesteguia S, Gaetano LC (2011). Highly specialized mammalian skulls from the Late Cretaceous of South America. Nature.

[22] Luo Z-X, Gatesy SM, Jenkins FA, Amaral WW, Shubin NH (2015). Mandibular and dental characteristics of Late Triassic mammaliaform *Haramiyavia* and their ramifications for basal mammal evolution. P Natl Acad Sci USA.

[23] Krause DW, Hoffmann S, Wible JR, Kirk EC, Schultz JA, von Koenigswald W, Groenke JR, Rossie JB, O’Connor PM, Seiffert ER, Dumont ER, Holloway WL, Rogers RR, Rahantarisoa LJ, Kemp AD, Andriamialison H (2014). First cranial remains of a gondwanatherian mammal reveal remarkable mosaicism. Nature.

[24] Zheng XT, Bi SD, Wang XL, Meng J (2013). A new arboreal haramiyid shows the diversity of crown mammals in the Jurassic period. Nature.

[25] Bi SD, Wang YQ, Guan J, Sheng X, Meng J (2014). Three new Jurassic euharamiyidan species reinforce early divergence of mammals. Nature.

[26] Averianov AO, Lopatin AV (2014). On the phylogenetic position of monotremes (Mammalia, Monotremata). Paleontol J.

[27] Meng J, Hu YM, Wang YQ, Li CK (2003). The ossified Meckel’s cartilage and internal groove in Mesozoic mammaliaforms: implications to origin of the definitive mammalian middle ear. Zool J Linn Soc-Lond.

[28] Tate A. Your Inner Reptile, Your Inner Fish, Public Broadcasting Service (PBS). 2014

[29] Hopson J, Rich T, Vickers-Rich P, Pamela G, Morton S (2009). Did the Cretaceous monotreme *Teinolophos trusleri* possess an internal mandibular trough for postdentary bones?. J Vertebr Paleontol.

[30] Rowe T, Rich TH, Vickers-Rich P, Springer M, Woodburne MO (2008). The oldest platypus and its bearing on divergence timing of the platypus and echidna clades. P Natl Acad Sci USA.

[31] Archer M, Flannery TF, Ritchie A, Molnar RE (1985). First Mesozoic mammal from Australia - an Early Cretaceous monotreme. Nature.

[32] Martin T, Rauhut OWM (2005). Mandible and dentition of *Asfaltomylos patagonicus* (Australosphenida, Mammalia) and the evolution of tribosphenic teeth. J Vertebr Paleontol.

[33] Zhou CF, Wu SY, Martin T, Luo Z-X (2013). A Jurassic mammaliaform and the earliest mammalian evolutionary adaptations. Nature.

[34] Rich TH, Vickers-Rich P, Constantine A, Flannery TF, Kool L, van Klaveren N (1997). A tribosphenic mammal from the Mesozoic of Australia. Science.

[35] Rich TR, Flannery TF, Trusler P, Kool L, van Klaveren NA, Vickers-Rich PP (2001). A second tribosphenic mammal from the Mesozoic of Australia. Rec Queen Victoria Mus.

[36] Reed DA, Iriarte-Diaz J, Diekwisch TGH (2016). A three dimensional free body analysis describing variation in the musculoskeletal configuration of the cynodont lower jaw. Evol Dev.

[37] Kielan-Jaworowska Z, Cifelli RL, Luo Z-X (2004). Mammals from the age of dinosaurs: Origins, evolution, and structure.

[38] Slater GJ (2015). Iterative adaptive radiations of fossil canids show no evidence for diversity-dependent trait evolution. P Natl Acad Sci USA.

[39] Rich TH, Hopson JA, Gill PG, Trusler P, Rogers-Davidson S, Morton S, Cifelli RL, Pickering D, Kool L, Siu K, Burgmann FA, Senden T, Evans AR, Wagstaff BE, Seegets-Villiers D, Corfe IJ, Flannery TF, Walker K, Musser AM, Archer M, Pian R, Vickers-Rich P (2016). The mandible and dentition of the Early Cretaceous monotreme *Teinolophos trusleri*. Alcheringa.

[40] Ji Q, Luo Z-X, Yuan C-X, Tabrum AR (2006). A swimming mammaliaform from the Middle Jurassic and ecomorphological diversification of early mammals. Science.

[41] Ronquist F, Huelsenbeck JP (2003). MrBayes 3: Bayesian phylogenetic inference under mixed models. Bioinformatics.

[42] Ronquist F, Teslenko M, van der Mark P, Ayres DL, Darling A, Hohna S, Larget B, Liu L, Suchard MA, Huelsenbeck JP (2012). MrBayes 3.2: Efficient Bayesian phylogenetic inference and model choice across a large model space. Syst Biol.

[43] Rambaut A, Drummond AJ. Tracer v1.5. 2007. http://tree.bio.ed.ac.uk/software/tracer/.

[44] Wiens JJ (2003). Missing data, incomplete taxa, and phylogenetic accuracy. Syst Biol.

[45] Wu JH, Susko E, Roger AJ (2008). An independent heterotachy model and its implications for phylogeny and divergence time estimation. Mol Phylogenet Evol.

[46] Woodburne MO, Rich TH, Springer MS (2003). The evolution of tribospheny and the antiquity of mammalian clades. Mol Phylogenet Evol.

[47] Phillips MJ, Pratt RC (2008). Family-level relationships among the Australasian marsupial “herbivores” (Diprotodontia : Koala, wombats, kangaroos and possums). Mol Phylogenet Evol.

[48] Kermack KA, Mussett F, Rigney HW (1973). The lower jaw of *Morganucodon*. Zool J Linn Soc-Lond.

