# Supplementary material for: Resolving the evolution of the mammalian middle ear using Bayesian inference
Source: Front Zool. 2016 Aug 24;13(1):39. doi: 10.1186/s12983-016-0171-z (PMC4997658; doi:10.1186/s12983-016-0171-z)
Supplement: Additional file 1: Figure S1. — Bayesian inference phylogeny using Rougier et al.’s [21] dataset when cheek tooth characters were excluded. This analysis clarified the placement of the early “symmetrodont” Kuehneotherium outside the mammalian crown. Figure S2. Bayesian inference phylogeny for Luo et al.’s [22] dataset, but with cheek tooth characters excluded from. The “pseudotribosphenic” shuotheriids (purple) fell outside crown mammals and the placement of the enigmatic, fossorial Fruitafossor (yellow) was clarified as grouping closer to therians than to monotremes (green). Table S1. Posterior probabilities. Description: A: Shuotheriidae allowed to group with australosphenidans. B: Constraining Shuotheriidae to fall outside Mammalia. C: Constraining monophyly of the multicuspate haramiyidans and multituberculates. D: Forces Kuehneotherium outside Mammalia. E: Allows the unstable Kuehneotherium to fall freely in the phylogeny. 1: Alternative analyses including Aukstribosphenos and Steropodon with postdentary trough coded as absent. (DOCX 231 kb) [file 12983_2016_171_MOESM1_ESM.docx]

**Fig. S1** Bayesian inference phylogeny using Rougier *et al*.’s [21] dataset when cheek tooth characters were excluded. This analysis clarified the placement of the early “symmetrodont” *Kuehneotherium* outside the mammalian crown.

**Fig. S2** Bayesian inference phylogeny for Luo *et al*.’s [22] dataset, but with cheek tooth characters excluded from. The “pseudotribosphenic” shuotheriids (purple) fell outside crown mammals and the placement of the enigmatic, fossorial *Fruitafossor* (yellow) was clarified as grouping closer to therians than to monotremes (green).

**Table S1** Posterior probabilities. Description: A: Shuotheriidae allowed to group with australosphenidans. B: Constraining Shuotheriidae to fall outside Mammalia. C: Constraining monophyly of the multicuspate haramiyidans and multituberculates. D: Forces *Kuehneotherium* outside Mammalia. E: Allows the unstable *Kuehneotherium* to fall freely in the phylogeny. 1: Alternative analyses including *Aukstribosphenos* and *Steropodon* with postdentary trough coded as absent.

|  | Luo *et a*l. (2015) | |  |  |  |  | Rougier *et a*l. (2011) | |  |  |
| --- | --- | --- | --- | --- | --- | --- | --- | --- | --- | --- |
| Post-dentary trough: (0) present, (1) absent | | | | | | | | | | |
|  | A | A1 | B | B1 | C | C1 | D | D1 | E | E1 |
| p(0) Mammalia | 0.85 | 0.86 | 0.59 | 0.61 | 0.95 | 0.95 | 0.98 | 0.98 | 0.94 | 0.95 |
| p(1) Mammalia | 0.15 | 0.14 | 0.41 | 0.39 | 0.05 | 0.05 | 0.02 | 0.02 | 0.06 | 0.05 |
| p(0) Theriiformes+*Fruitafosso*r+Euharamiyida | 0.13 | 0.11 | 0.09 | 0.08 | 0.86 | 0.86 | NA | NA | NA | NA |
| p(1) Theriiformes+*Fruitafossor*+Euharamiyida | 0.87 | 0.89 | 0.91 | 0.92 | 0.14 | 0.14 | NA | NA | NA | NA |
| p(0) Theriiformes | 0.00 | 0.00 | 0.00 | 0.00 | 0.00 | 0.00 | 0.02 | 0.02 | 0.02 | 0.02 |
| p(1) Theriiformes | 1.00 | 1.00 | 1.00 | 1.00 | 1.00 | 1.00 | 0.98 | 0.98 | 0.98 | 0.98 |
| p(0) Mammaliaformes | 1.00 | 1.00 | 1.00 | 1.00 | 1.00 | 1.00 | 1.00 | 1.00 | 1.00 | 1.00 |
| p(1) Mammaliaformes | 0.00 | 0.00 | 0.00 | 0.00 | 0.00 | 0.00 | 0.00 | 0.00 | 0.00 | 0.00 |
| p(0) Cladotheria | 0.00 | 0.00 | 0.00 | 0.00 | 0.00 | 0.00 | 0.00 | 0.00 | 0.00 | 0.00 |
| p(1) Cladotheria | 1.00 | 1.00 | 1.00 | 1.00 | 1.00 | 1.00 | 1.00 | 1.00 | 1.00 | 1.00 |
| p(0) Mammalia+*Hadrocodium* | 0.99 | 0.99 | 0.97 | 0.98 | 0.99 | 0.99 | NA | NA | NA | NA |
| p(1) Mammalia+*Hadrocodium* | 0.01 | 0.01 | 0.03 | 0.02 | 0.01 | 0.01 | NA | NA | NA | NA |
| p(0) Crown Monotremata | 0.00 | 0.00 | 0.00 | 0.00 | 0.00 | 0.00 | NA | NA | NA | NA |
| p(1) Crown Monotremata | 1.00 | 1.00 | 1.00 | 1.00 | 1.00 | 1.00 | NA | NA | NA | NA |
| p(0) Monotremata | 0.00 | 0.00 | 0.00 | 0.00 | 0.01 | 0.00 | 0.00 | 0.00 | 0.00 | 0.00 |
| p(1) Monotremata | 1.00 | 1.00 | 1.00 | 1.00 | 0.99 | 1.00 | 1.00 | 1.00 | 1.00 | 1.00 |
| p(0) Australian Australosphenida | 0.06 | 0.00 | 0.04 | 0.00 | 0.06 | 0.00 | 0.07 | 0.06 | 0.07 | 0.06 |
| p(1) Australian Australosphenida | 0.94 | 1.00 | 0.96 | 1.00 | 0.94 | 1.00 | 0.93 | 0.94 | 0.93 | 0.94 |
| p(0) Multituberculata | 0.00 | 0.00 | 0.00 | 0.00 | 0.00 | 0.00 | NA | NA | NA | NA |
| p(1) Multituberculata | 1.00 | 1.00 | 1.00 | 1.00 | 1.00 | 1.00 | NA | NA | NA | NA |
| p(0) Australosphenida | 0.94 | 0.94 | 0.64 | 0.66 | 0.92 | 0.93 | 0.92 | 0.92 | 0.92 | 0.92 |
| p(1) Australosphenida | 0.06 | 0.06 | 0.36 | 0.34 | 0.08 | 0.07 | 0.08 | 0.08 | 0.08 | 0.08 |
| p(0) Tribosphenida | 0.00 | 0.00 | 0.00 | 0.00 | 0.00 | 0.00 | 0.00 | 0.00 | 0.00 | 0.00 |
| p(1) Tribosphenida | 1.00 | 1.00 | 1.00 | 1.00 | 1.00 | 1.00 | 1.00 | 1.00 | 1.00 | 1.00 |
| p(0) Theria | 0.00 | 0.00 | 0.00 | 0.00 | 0.00 | 0.00 | 0.00 | 0.00 | 0.00 | 0.00 |
| p(1) Theria | 1.00 | 1.00 | 1.00 | 1.00 | 1.00 | 1.00 | 1.00 | 1.00 | 1.00 | 1.00 |
| p(0) Multituberculata+*Tinodon*+Trechnotheria | 0.00 | 0.00 | 0.00 | 0.00 | NA | NA | NA | NA | NA | NA |
| p(1) Multituberculata+*Tinodon*+Trechnotheria | 1.00 | 1.00 | 1.00 | 1.00 | NA | NA | NA | NA | NA | NA |
| p(0) Trechnotheria | 0.00 | 0.00 | 0.00 | 0.00 | 0.00 | 0.00 | 0.00 | 0.00 | 0.00 | 0.00 |
| p(1) Trechnotheria | 1.00 | 1.00 | 1.00 | 1.00 | 1.00 | 1.00 | 1.00 | 1.00 | 1.00 | 1.00 |
| p(0) Euharamiyida + Multituberculata | NA | NA | NA | NA | 0.30 | 0.27 | NA | NA | NA | NA |
| p(1) Euharamiyida + Multituberculata | NA | NA | NA | NA | 0.70 | 0.73 | NA | NA | NA | NA |
| p(0) Haramiyida+Euharamiyida+Multituberculata | NA | NA | NA | NA | 0.93 | 0.93 | NA | NA | NA | NA |
| p(1) Haramiyida+Euharamiyida+Multituberculata | NA | NA | NA | NA | 0.07 | 0.07 | NA | NA | NA | NA |

| **Meckelian groove in adults: (0) developed, (1) vestigial or absent** | | | | | | | | | | |
| --- | --- | --- | --- | --- | --- | --- | --- | --- | --- | --- |
|  | A | A1 | B | B1 | C | C1 | D | D1 | E | E1 |
| p(0) Mammalia | 0.95 | 0.96 | 0.94 | 0.95 | 0.97 | 0.97 | 0.99 | 0.99 | 0.99 | 0.99 |
| p(1) Mammalia | 0.05 | 0.04 | 0.06 | 0.05 | 0.03 | 0.03 | 0.01 | 0.01 | 0.01 | 0.01 |
| p(0) Theriiformes+*Fruitafossor*+Euharamiyida | 0.94 | 0.95 | 0.93 | 0.94 | 0.96 | 0.97 | NA | NA | NA | NA |
| p(1) Theriiformes+*Fruitafossor*+Euharamiyida | 0.06 | 0.05 | 0.07 | 0.06 | 0.04 | 0.03 | NA | NA | NA | NA |
| p(0) Theriiformes | 0.98 | 0.97 | 0.97 | 0.97 | 0.97 | 0.96 | 0.99 | 0.99 | 1.00 | 0.99 |
| p(1) Theriiformes | 0.02 | 0.03 | 0.03 | 0.03 | 0.03 | 0.04 | 0.01 | 0.01 | 0.00 | 0.01 |
| p(0) Mammaliaformes | 0.99 | 0.99 | 0.99 | 0.99 | 1.00 | 1.00 | 1.00 | 1.00 | 1.00 | 1.00 |
| p(1) Mammaliaformes | 0.01 | 0.01 | 0.01 | 0.01 | 0.00 | 0.00 | 0.00 | 0.00 | 0.00 | 0.00 |
| p(0) Cladotheria | 1.00 | 1.00 | 1.00 | 1.00 | 1.00 | 1.00 | 0.99 | 1.00 | 1.00 | 1.00 |
| p(1) Cladotheria | 0.00 | 0.00 | 0.00 | 0.00 | 0.00 | 0.00 | 0.01 | 0.00 | 0.00 | 0.00 |
| p(0) Mammalia+*Hadrocodium* | 0.97 | 0.97 | 0.97 | 0.97 | 0.98 | 0.98 | NA | NA | NA | NA |
| p(1) Mammalia+*Hadrocodium* | 0.03 | 0.03 | 0.03 | 0.03 | 0.02 | 0.02 | NA | NA | NA | NA |
| p(0) Crown Monotremata | 0.03 | 0.03 | 0.03 | 0.03 | 0.02 | 0.02 | NA | NA | NA | NA |
| p(1) Crown Monotremata | 0.97 | 0.97 | 0.97 | 0.97 | 0.98 | 0.98 | NA | NA | NA | NA |
| p(0) Monotremata | 0.87 | 0.88 | 0.87 | 0.88 | 0.87 | 0.89 | 0.90 | 0.90 | 0.90 | 0.90 |
| p(1) Monotremata | 0.13 | 0.12 | 0.13 | 0.12 | 0.13 | 0.11 | 0.10 | 0.10 | 0.10 | 0.10 |
| p(0) Australian Australosphenida | 0.99 | 1.00 | 0.99 | 0.99 | 0.99 | 1.00 | 0.98 | 0.98 | 0.98 | 0.98 |
| p(1) Australian Australosphenida | 0.01 | 0.00 | 0.01 | 0.01 | 0.01 | 0.00 | 0.02 | 0.02 | 0.02 | 0.02 |
| p(0) Multituberculata | 0.01 | 0.01 | 0.01 | 0.01 | 0.00 | 0.00 | NA | NA | NA | NA |
| p(1) Multituberculata | 0.99 | 0.99 | 0.99 | 0.99 | 1.00 | 1.00 | NA | NA | NA | NA |
| p(0) Australosphenida | 0.86 | 0.88 | 0.82 | 0.83 | 0.86 | 0.88 | 0.97 | 0.97 | 0.97 | 0.97 |
| p(1) Australosphenida | 0.14 | 0.12 | 0.18 | 0.17 | 0.14 | 0.12 | 0.03 | 0.03 | 0.03 | 0.03 |
| p(0) Tribosphenida | 0.98 | 0.98 | 0.98 | 0.98 | 0.98 | 0.99 | 0.97 | 0.97 | 0.97 | 0.97 |
| p(1) Tribosphenida | 0.02 | 0.02 | 0.02 | 0.02 | 0.02 | 0.01 | 0.03 | 0.03 | 0.03 | 0.03 |
| p(0) Theria | 0.51 | 0.52 | 0.52 | 0.52 | 0.51 | 0.51 | 0.70 | 0.67 | 0.71 | 0.66 |
| p(1) Theria | 0.49 | 0.48 | 0.48 | 0.48 | 0.49 | 0.49 | 0.30 | 0.33 | 0.29 | 0.34 |
| p(0) Multituberculata+*Tinodon*+Trechnotheria | 0.89 | 0.90 | 0.88 | 0.90 | NA | NA | NA | NA | NA | NA |
| p(1) Multituberculata+*Tinodon*+Trechnotheria | 0.11 | 0.10 | 0.12 | 0.10 | NA | NA | NA | NA | NA | NA |
| p(0) Trechnotheria | 0.99 | 0.99 | 0.99 | 0.99 | 0.99 | 0.99 | 1.00 | 0.99 | 0.99 | 0.99 |
| p(1) Trechnotheria | 0.01 | 0.01 | 0.01 | 0.01 | 0.01 | 0.01 | 0.00 | 0.01 | 0.01 | 0.01 |
| p(0) Euharamiyida + Multituberculata | NA | NA | NA | NA | 0.45 | 0.43 | NA | NA | NA | NA |
| p(1) Euharamiyida + Multituberculata | NA | NA | NA | NA | 0.55 | 0.57 | NA | NA | NA | NA |
| p(0) Haramiyida+Euharamiyida+Multituberculata | NA | NA | NA | NA | 0.94 | 0.95 | NA | NA | NA | NA |
| p(1) Haramiyida+Euharamiyida+Multituberculata | NA | NA | NA | NA | 0.06 | 0.05 | NA | NA | NA | NA |
